# The effectiveness of spinal manipulative therapy procedures for spine pain: protocol for a systematic review and network meta-analysis

**DOI:** 10.1186/s12998-023-00487-z

**Published:** 2023-05-24

**Authors:** Casper G. Nim, Sasha L. Aspinall, Chad E. Cook, Leticia A. Corrêa, Megan Donaldson, Aron S. Downie, Steen Harsted, Jan Hartvigsen, Hazel J. Jenkins, David McNaughton, Luana Nyirö, Stephen M. Perle, Eric J. Roseen, James J. Young, Anika Young, Gong-He Zhao, Carsten B. Juhl

**Affiliations:** 1 Medical Research Unit, Spine Centre of Southern Denmark, Lillebaelt Hospital, University Hospital of Southern Denmark, Østre Hougvej 55, Middelfart, Denmark; 2 Department of Regional Health Research, University of Southern Denmark, Odense, Denmark; 3 Department of Sports Science and Clinical Biomechanics, Center for Muscle and Joint Health, University of Southern Denmark, Odense, Denmark; 4 School of Allied Health, College of Health and Education, Murdoch University, Murdoch, Australia; 5 Department of Orthopaedics, Duke University, Durham, NC USA; 6 Duke Clinical Research Institute, Duke University, Durham, NC USA; 7 Department of Population Health Sciences, Duke University, Durham, NC USA; 8 Department of Chiropractic, Macquarie University, Macquarie Park, Australia; 9 Department of Rehabilitation Sciences, Medical University of South Carolina, Charleston, SC USA; 10 Chiropractic Knowledge Hub, Odense, Denmark; 11 School of Psychological Sciences, Macquarie University, Macquarie Park, Australia; 12 Department of Chiropractic Medicine, Balgrist University Hospital, University of Zurich, Zurich, Switzerland; 13 Big Data Interrogation Group, AECC University College, Bournemouth, UK; 14 Section of General Internal Medicine, Department of Medicine, Chobanian and Avedisian School of Medicine and Boston Medical Center, Boston University, Boston, MA USA; 15 Schroeder Arthritis Institute, Krembil Research Institute, University Health Network, Toronto, Canada; 16 School of Sport Science, Beijing Sport University, Beijing, China; 17 Department of Physiotherapy and Occupational Therapy, Copenhagen University Hospital, Copenhagen, Denmark

## Abstract

**Background:**

Spinal manipulative therapy (SMT) is a guideline-recommended treatment option for spinal pain. The recommendation is based on multiple systematic reviews. However, these reviews fail to consider that clinical effects may depend on SMT “application procedures” (i.e., how and where SMT is applied). Using network meta-analyses, we aim to investigate which SMT “application procedures” have the greatest magnitude of clinical effectiveness for reducing pain and disability, for any spinal complaint, at short-term and long-term follow-up. We will compare application procedural parameters by classifying the thrust application technique and the application site (patient positioning, assisted, vertebral target, region target, Technique name, forces, and vectors, application site selection approach and rationale) against: 1. Waiting list/no treatment; 2. Sham interventions not resembling SMT (e.g., detuned ultrasound); 3. Sham interventions resembling SMT; 4. Other therapies not recommended in clinical practice guidelines; and 5. Other therapies recommended in clinical practice guidelines. Secondly, we will examine how contextual elements, including procedural fidelity (whether the SMT was delivered as planned) and clinical applicability (whether the SMT is similar to clinical practice) of the SMT.

**Methods:**

We will include randomized controlled trials (RCT) found through three search strategies, (i) exploratory, (ii) systematic, and (iii) other known sources. We define SMT as a high-velocity low-amplitude thrust or grade V mobilization. Eligibility is any RCT assessing SMT against any other type of SMT, any other active or sham intervention, or no treatment control on adult patients with pain in any spinal region. The RCTs must report on continuous pain intensity and/or disability outcomes. Two authors will independently review title and abstract screening, full-text screening, and data extraction. Spinal manipulative therapy techniques will be classified according to the technique application and choice of application sites. We will conduct a network-meta analysis using a frequentist approach and multiple subgroup and sensitivity analyses.

**Discussion:**

This will be the most extensive review of thrust SMT to date, and will allow us to estimate the importance of different SMT application procedures used in clinical practice and taught across educational settings. Thus, the results are applicable to clinical practice, educational settings, and research studies.

*PROSPERO registration*: CRD42022375836.

**Supplementary Information:**

The online version contains supplementary material available at 10.1186/s12998-023-00487-z.

## Background

Spinal manipulative therapy (SMT) is defined as a high-velocity, low-amplitude (HVLA) thrust intended to increase the mobility of a specific joint in the spine, improve function, and decrease pain [1]. It has been used for centuries and popularized by medical, allied health, and complementary and integrative health professions since the beginning of the twentieth century [2, 3]. Today, it is a common approach used to manage spine pain (i.e., cervical, lumbar, and thoracic spine with or without radiating pain) across multiple regulated healthcare professions (e.g., chiropractic, physiotherapy, osteopathy) [3, 4], and SMT is generally recommended in clinical guidelines as a first-line or adjunct therapy when treating spinal pain conditions [5]. Recommendations from systematic reviews and meta-analyses outline that the effect of SMT on pain intensity and disability is comparable to other recommended therapies (e.g., education and exercise) [6–10].

However, when examining individual studies, there is significant variation in outcomes across studies, suggesting that the clinical effect may differ between the disparate forms of SMT or that moderating factors such as contextual elements are notably different among studies [6–10]. Arguably, the SMT thrust is not a “one size fits all” approach. In fact, the thrust can be classified by several application techniques and applied at numerous sites across the spine. We label these different approaches as “application procedures,” which likely differ due to different teaching philosophies both across professions (e.g., chiropractic and physiotherapy) and within professions (e.g., in chiropractic: Gonstead and Upper Cervical Technique) [11]. Further, the application procedures can also be applied manually or assisted by an instrument (e.g., Activator™) [12]. There are various ways in which clinicians determine the application site, including pain provocation maneuvers, range of motion deficits, and palpation for perceived joint stiffness or “dysfunction” [13]. Contextual elements of the study design may also differ, and research and clinical practice are not always coherent, and *procedural fidelity* (e.g., unclear treatment protocol or difficulty adhering to it) and how *clinically applicable* the SMT is (i.e., the SMT being provided is comparable to clinical practice). All of the above may influence effectiveness in SMT studies [7, 14].

### Rationale and aim

Published reviews have not evaluated whether the clinical effectiveness of SMT varies by reported application procedures, fidelity to those procedures, or the context in which the SMT is applied [6–10]. Examining these more carefully may help explain the variations reported in previous reviews. This can be achieved through network meta-analysis procedures [15]. The network meta-analysis makes investigating different SMT application procedures’ direct and indirect effects possible. Explaining the variability may also reveal latent differences in clinical effects, which in turn can help clinicians adopt the most appropriate SMT application procedures. Moreover, the results can aid educational institutions in improving their curricula by teaching the most effective techniques or, conversely, the easiest, most preferable, and safest procedures, if nothing is superior.

We aim to conduct a systematic review and network meta-analysis to investigate which SMT application procedures have the greatest magnitude of clinical effectiveness for reducing pain and disability, for any spinal complaint, at short-term and long-term follow-up. We will investigate application procedural parameters classifying the thrust application technique and the application site against:

1. Waiting list/no treatment
2. Sham interventions not resembling SMT (e.g., detuned ultrasound)
3. Sham interventions resembling SMT
4. Other therapies not recommended in guidelines
5. Other therapies recommended in clinical practice guidelines

Secondly, we will examine how contextual elements including procedural fidelity and clinical applicability of the SMT impacts the effectiveness.

## Methods

This systematic review and network meta-analysis protocol is reported according to the *Preferred Reporting Items for Systematic reviews and Meta-Analyses for Protocols* (PRISMA-P) [16]. The systematic review and network meta-analysis will be reported according to the *Preferred Reporting items of Systematic reviews and Meta-Analyses for Network Meta-Analyses* (PRISMA-NMA) [17].

### Protocol and registration

The study was prospectively registered through PROSPERO (CRD42022375836) before initiating the search and the protocol was submitted for publication before extracting the data set and beginning data analysis.

### Eligibility criteria

We will include any randomized controlled trial assessing SMT against any other type of SMT, any other intervention, sham intervention, or no treatment control (e.g., waiting list). The studies must include participants where the majority are 18 years of age or over and none aged under 16, and report on pain intensity and/or disability outcomes. Patient-reported measures are limited to continuous scales for any non-specific, degenerative, or inflammatory pain condition (e.g., not cancer pain) in the cervical, thoracic, or lumbosacral spine of any episode/pain duration.

We will exclude studies that assess only mobilizations where an HVLA thrust is not provided (as per Maitland criteria) [18] and studies where the effects of SMT are not directly measurable (i.e., where SMT was delivered as part of a multimodal care package with the same co-interventions not delivered in the other arms).

### Information sources

We will use three sources to obtain the references included in the study selection process.

#### Exploratory approaches

This approach uses previously published data [19]. Systematic reviews investigating the effect of SMT on any patient-reported outcome were retrieved from PubMed and Epistemonikos (a health evidence database including more than 478,000 systematic reviews, making it the largest source of systematic reviews relevant for health-decision making—https://www.epistemonikos.org) for all entries up to February 25th, 2022 [20].

For PubMed, the search term “Musculoskeletal manipulations” [MeSH] and the filter “Systematic reviews” was applied. For Epistemonikos, a search by title or abstract using the search terms combined with the Boolean operators (musculoskeletal OR spinal*) AND (manipulation* OR adjust* OR chiropract*) and filtered for systematic reviews. Systematic reviews were excluded if they (a) did not investigate effect of SMT, (b) did not include patient-reported outcomes, (c) focused on cost-effectiveness, and (d) focused on adverse events only. Two researchers performed the title and abstract screening process independently, and conflicts were solved by discussion. The initial search yielded 1256 results, of which 128 were duplicates. After title and abstract screening, 314 systematic reviews were eligible for full-text review, and 85 systematic reviews were included and reported on 442 distinct references to be eligible in the study selection process.

#### Systematic search

As our exploratory approach identified 442 references across 85 systematic reviews until early 2022, we do not expect to find many trials not included in this list. Therefore, we will limit our systematic search from January 1st, 2018, to December 2nd, 2022, to ensure the identification of more recent trials. We will systematically search in MEDLINE, EMBASE, Cochrane Central Register of Controlled Trials (CENTRAL), Physiotherapy Evidence Database (PEDro), and Index to Chiropractic Literature (Additional file 1: Appendix 1). We intend to update this search for any secondary analyses.

#### Other sources

References will be added from newer known systematic reviews, not included in the exploratory search and systematic reviews found through CENTRAL [6–8, 8–10, 21–23]. If the authors become aware of newer studies during the review process, these will also be included.

### Study selection

All references from the three sources described above will be imported into Covidence, where duplicates will be removed automatically [24]. Two reviewers (any pair of CGN, SLA, AD, SH, HJJ, LN, JJY or SMP) will screen the title and abstracts of the remaining references independently. CGN and SLA will discuss any conflicts until an agreement is reached, if consensus cannot be reached a third author will resolve any conflicts (either CEC, JH, SMP, CBJ). The full text of all eligible studies will be retrieved and reviewed independently by two reviewers (any pair of CGN, SLA, LAC, MD AD, SH, HJJ, DM, LN, EJR, AY, JJY, GHZ) using the same approach.

Once all relevant references are selected, “Research Rabbit” [25] will be used to identify any additional relevant references. CGN will perform this step, and if additional references are suggested for inclusion, this will be determined in consensus with SLA.

#### Data collection and SMT classification process

Two reviewers (CGN, SLA, LAC, MD AD, SH, HJJ, DM, LN, EJR, AY, JJY, GHZ) will independently extract data using the Covidence data-extraction module 2.0. Data will be extracted on:

- Identifier (author, year of publication, country of origin, study setting, number of participants)
- Patient population (region of the spinal pain, age, baseline pain, duration of pain, recruitment source)
- SMT procedures (i.e., patient positioning, instrument or drop-piece assisted, vertebral target, region target, Technique name, forces, and vectors, application site selection approach and rationale)
- Treatment dosage (session length, frequency, and duration)
- Clinician (profession, years of experience)
- Comparator details
- Details on adjunct intervention(s) provided in both groups
- Outcome measures and follow-ups for self-reported pain intensity and disability
- Average and variability scores on pain intensity and disability outcomes at baseline and two follow-up time points; closest to the end of treatment and closest to 12 months: The selection of these time points was based on several factors. Firstly, we chose immediate post-treatment as it allows for a direct comparison of differences across SMT procedures; and secondly, we opted a long follow-up period to assess whether the SMT procedures resulted in long-term benefits.
- For sham procedures; if the sham is listed as valid or tested if it is inert on the subjects, and if participant blinding was assessed as adequate, inadequate, or not assessed

#### SMT classification

We will classify each SMT arm across two different classification systems performed by two independent reviewers. A priori, we developed a list of classifications based on the “Consensus on Interventions Reporting Criteria List for Spinal Manipulative Therapy” by Groeneweg et al. [26]. However, the approach will be iterative and data-driven so that categories may be added or some may be merged into smaller groups. There will be an option to rate each classification as “not specified” if insufficient information is reported in the study.

The classifications and procedures are:

I. The technique applied is assessed according to the prior categorization work [26–28]. Each SMT arm is categorized using the following items: patient position, use of assistance, specific vertebral target, region/s targeted, and specific techniques (Additional file 1: Appendix 2).
II. The choice of application site is assessed based on an adapted version of the work by Triano et al. [13] and was categorized based on the selection approach, technique approach, and the rationale for the selection approach (Additional file 1: Appendix 2).

We chose these two classification systems as they arguably reflect the most fundamental aspects of the SMT (i.e., every clinician has to select an appropriate SMT technique AND apply it to a target spinal site). Additionally, these two aspects are consistent across manual therapy professions and are central when teaching SMT.

#### Procedural fidelity within individual studies

Procedural fidelity will be based on selected criteria by Borelli et al. [14] and will focus on the fidelity of treatment delivery. We will simplify the assessment to include the following items:

I. Included method to ensure that the content of the intervention was being delivered as specified (e.g., checklist, computer program)
II. Included method to ensure that the dose of the intervention was being delivered as specified (e.g., records number of contact minutes)
III. Included mechanism to assess if the provider actually adhered to the intervention plan (e.g., audiotape, observation, self-report of provider, exit interview with participant)
IV. Used a treatment manual/protocol

Each item will be scored as “Yes’’ or” No” or “Not reported” by two reviewers independently. The results will be tabulated by the SMT intervention arm.

#### Clinically applicable SMT within individual studies

The SMT protocol for each study will be further scored for clinical applicability of the SMT procedure based on the CIRCLe-SMT checklist [26] with indicators for being applicable as shown below:

I. Relevant Clinician/physician (Yes if: Chiropractor, Physiotherapist, Osteopath, Medical doctor, and Manual therapist with different backgrounds)
II. Treatment setting (Yes if: any clinical setting)
III. Session length (Yes if: ≥ 10 min)
IV. Session frequency (Yes if: ≥ two times per week)
V. Duration of treatment (Yes if: ≥ 2 weeks)
VI. Tailoring the intervention to individual participants (Yes if: yes)
VII. Treatment region (Yes if: includes the region of pain)
VIII. Segment specific (Yes if: yes)

Each item is assessed as “applicable” if it matches the above-stated criteria and “Not applicable” if it does not match the above-stated criteria. Each study’s clinical applicability will be categorized using the scoring of the items by at least two reviewers (all with a background as chiropractor or physiotherapist and have more than five years of clinical experience) until consensus is reached, if consensus cannot be reached a third senior author (SMP, CEC, JH, CBJ) will finalize the decision.

#### Comparator classification

Wait-list control/No treatment is any control that does not provide participants with an active intervention (e.g., studies that provide participants with a pamphlet).

Sham interventions not resembling SMT is based on (i) described as a sham/placebo and/or intended to blind participants, and (ii) not replicate the positioning/procedure of SMT, e.g. detuned ultrasound, sham “functional” techniques involving hand contact with no positioning.

Sham interventions resembling SMT is based on (i) described as a sham/placebo and/or patients are intended to be blinded, and (ii) replicate the procedure/positioning/context of the SMT being delivered without a thrust (e.g., passive positioning or joint pre-tension without thrusting, instrument delivering minimal thrust (if being compared to thrust instrumentation), drop-piece without joint thrust).

Recommended and non-recommended therapies is based on European national guidelines using the synthesized list from Corp et al. and rated as Recommended, Not-recommended, or mixed [5]. Any other interventions not captured by the criteria above will be listed as non-recommended.

#### Risk of bias within individual studies

Two reviewers will independently assess the risk of bias using Cochrane’s Risk of Bias Tool version 2 [29], with a final agreement decided through discussion.

We will define a study as having high risk of bias if any domain is scored as high risk or three or more domains are scored as having some concerns. The study will only be assessed as having low risk of bias if all domains are scored as low risk.

#### Limiting bias in the assessment approach

If any authors is also an author of a study that is assessed in this review (from study selection to risk of bias assessment), assessment of that study will be conducted by another member of the review team in order to limit potential for bias. Additionally, to ensure consistency in the data extraction and risk of bias assessment, all conflicts are handled by CGN in discussion with SLA, if a conflict cannot be resolved a third senior author (SMP, CEC, JH, CBJ) will finalize the decision.

## Statistical and analytical procedures

### Geometry of the network

For each outcome (pain and disability), we will conduct separate network meta-analyses for the thrust technique and for the thrust application site. Each will be presented graphically with a network plot illustrating the number of comparisons in the two SMT classifications. This will allow for comparisons between various aspects of the SMT procedures (e.g., prone versus supine, manual versus instrument assisted, or treating a symptomatic versus a non-symptomatic region), and aspects of the choice of application site (e.g., clinician-selected versus predetermined, or selected based on complaint history versus palpation). The analyses will be conducted in R vers. 4.2 using the *netmeta package* [30].

## Summary measures

The effect of the intervention (pain intensity and disability) will be calculated as the standardized mean difference (SMD) adjusted to Hedges g on differences in outcome between different SMT types according to the classification, allowing pooling of outcomes within the outcome domain. The SMD will be estimated as the difference in unadjusted mean change from baseline to after the intervention between each intervention and comparison groups divided by the pooled standard deviation (SD) per study comparison. If the SD is not available, it will be estimated from standard error (SE), the confidence interval (CI), the p-value, or other methods recommended by the Cochrane Handbook [31]. Standardized mean differences will be adjusted to Hedges g to account for slightly overestimated effects in smaller studies. The SMD will be reported as the numeric value and then clinically interpreted as originally proposed by Cohen (e.g., small effect = 0.2, moderate effect = 0.5 and large effect = 0.8 [31]). Meta-analyses will be performed using a random effects model with heterogeneity variance estimation using restricted maximum likelihood (REML) due to expected heterogeneity in participants, interventions, and outcome measures.

### Planned methods of analysis

We will perform a network meta-analysis based on the direct and indirect effect size estimates between the different SMT classifications. Network meta-analyses are a generalization of meta-analysis methods that allow comparisons of interventions not directly comparable within individual primary trials. Studies with more than one intervention group will be split in two, dividing the number of participants in the control group by the number of comparisons, thereby increasing the SE to avoid double counting. We will calculate the probability of each type of SMT being the most effective (highest SMD). We will report on an effect matrix showing the different comparisons in columns and rows. Next, probability values will be summarized and reported as the surface under the cumulative ranking (SUCRA) [32]. When SUCRA = 1, this SMT classification consistently ranks first and thereby is considered the most effective, and 0 if it consistently ranks last and the least effective in changing the outcomes of interest.

#### Assessment of inconsistency

The proportion of the variance in observed effect is due to variance in true effects rather than sampling error (hereby termed heterogeneity) will be examined for each pair-wise comparison as between-study variance (τ^2^) and calculated as the I^2^ statistic measuring the proportion of variation (i.e., inconsistency) in the networks.

A network meta-analysis forest plot will be produced, and inconsistency will be evaluated visually on the difference between estimates based on the direct and indirect estimates of the effect. We will check the overall model for consistency and apply an F-test to evaluate consistency.

#### Risk of bias across studies

We will produce a summary of the overall scores and highlight the main sources of risk of bias.

#### Confidence of findings

We will use The Confidence in Network Meta-Analysis (CINeMA) approach which is based on the GRADE framework [33]. The CINeMA covers 6 domains: (i) within-study bias (referring to the impact of risk of bias in the included studies), (ii) reporting bias (referring to publication and other reporting bias), (iii) indirectness, (iv) imprecision, (v) heterogeneity, and (vi) incoherence. Each domain is scored as no concerns, some concerns, or major concerns. The final judgments will be summarized across domains as the confidence for each relative treatment effect (i.e., very low, low, moderate, or high). Two reviewers will independently score each treatment effect, and final consensus will be based on discussion.

#### Procedural fidelity

We will compare effects when including versus excluding studies lacking procedural fidelity (i.e., scoring “No”); analysis will be repeated for each of the four items and determined if studies with better procedural fidelity influence treatment effect and ranking. Categorization of procedural fidelity will be based on the data availability.

#### Clinically applicable

We will compare effects when including versus excluding studies deemed not being clinically applicable to determine whether this affects treatment effect and ranking. Categorization of whether an SMT arm is clinically applicable will be based on the data availability and will reflect: efficacy versus effectiveness, a similar context for application as seen in a clinical setting, a similar communication context when discussing the purpose and expected response with the patient.

### Additional analyses

#### Subgroup analyses

Analyses will be repeated and subgrouped by the following:

##### Clinician

- Profession: chiropractors, physiotherapist (or physical therapist or manual physical therapist), osteopaths, medical doctor, massage therapist, and others.
- Median years of clinical experience: Under five years of experience, five or more years of experience, not specified, and not able to clarify.

##### Patient population

- Median age: young adults (< 40), middle age (40–60), older age (> 60), not specified, and not able to clarify
- Pain region: pelvis and lumbar, thoracic, and cervical spine
- Pain symptoms: Subgrouped by (i) mean pain duration: non-persistent (< 12 weeks) and persistent (≥ 12 weeks). (ii) mean pain intensity score at entry: low (rating < 6/10) and high (rating ≥ 6/10)

##### SMT dose

- Number of treatment sessions (n sessions)
- Treatment period (Duration between first and last session)
- Treatment time (Median treatment time)

#### Sensitivity analysis

##### Risk of bias

- Including versus excluding high risk of bias studies: does excluding high RoB studies influence treatment effect and ranking?

##### Sham interventions resembling SMT

- Including versus excluding sham SMT arms that are not reported as valid (either by reference or by testing the subjects), and based on whether participants were assessed as adequately blinded or not (either not adequately blinded or not assessed)

### Scientific publications

The analysis described above will provide the basis for at least three peer-reviewed scientific publications:

I: ***The effectiveness of spinal manipulative therapy procedures for spine pain: a systematic review and network meta-analysis***

This study will describe the main objectives stated in this protocol (i.e., The direct and indirect effects of different SMT application procedures on pain and disability outcomes compared with other interventions, sham interventions, and no treatment controls). Additionally, provide the above-stated subgroup -and sensitivity analyses: clinical-based subgroups (e.g., clinician and patient populations), SMT dose (e.g., sessions and numbers of thrust), and Risk of Bias (i.e., high versus low RoB).

II: ***Spinal manipulative thrust application procedures: An exploratory network meta-epidemiological study***

This study will report on how procedure fidelity and clinical applicability affect the results obtained and will provide the results on the above-stated characteristics not reported in the primary publication.

III: ***Spinal manipulative therapy: are the published effects explained by factors not related to the design***

This study will be explorative in nature and provide meta-regression analyses on non-SMT-related items. A-priori, we will investigate (i) time of publication (i.e., does the effect of SMT change over time), (ii) scientific journal (i.e., is the scientific journal associated with the effect size of SMT), (iii) country of study, and (iv) recruitment source.

## Discussion

This will be the most extensive review on thrust SMT to date and will allow us to estimate the importance of the different SMT application procedures used in clinical practice and educational settings. Thus, the results will aid clinicians and educators in optimizing treatment and teaching SMT consistently across different clinical populations. The results are also critical for mechanistic and translational research, as mechanistic research using animal models can provide precise thrust with a fixed force and vector to a standardized application site. If any such thrust and application sites are superior in human trials, this can be used to improve basic science and translational studies. Furthermore, the SMT research community can begin to explore the mechanisms behind different effects (if any) between different SMT application procedures. Finally, this data set will allow for collaboration across many interested research units and can provide the basis for multiple meta-regressions analyses.

## Supplementary Information

**Additional file 1**. The search strategy across databases.

## Data Availability

Not applicable.

## Abbreviations

SMT: Spinal manipulative therapy
RCT: Randomized controlled trial
HVLA: High-Velocity Low-Amplitude
PRISMA-P: Preferred Reporting Items for Systematic reviews and Meta-Analyses for Protocols
PRISMA-NMA: Preferred Reporting items of Systematic reviews and Meta-Analyses for Network Meta-Analyses
CENTRAL: The Cochrane Central Register of Controlled Trials
PEDRO: Physiotherapy Evidence Database
SMD: Standardized mean difference
SD: Standard deviation
SE: Standard error
REML: Random effect model
SUCRA: Surface under the cumulative ranking
CINeMA: Confidence in Network Meta-Analysis
