# Supplementary material for: The effectiveness of spinal manipulative therapy procedures for spine pain: protocol for a systematic review and network meta-analysis
Source: Chiropr Man Therap. 2023 May 24;31:14. doi: 10.1186/s12998-023-00487-z (PMC10210472; doi:10.1186/s12998-023-00487-z)
Supplement: Supplementary file 1 — Additional file 1. The search strategy across databases. [file 12998_2023_487_MOESM1_ESM.docx]

The effectiveness of spinal manipulative therapy procedures for spine pain: protocol for a systematic review and network meta-analysis analysis

# Appendix

## Appendix 1

The search strategy across databases

### MEDLINE (via Ovid), December 2nd, 2022

1 randomized controlled trial.pt. ()

2 controlled clinical trial.pt. ()

3 randomized.ab. ()

4 placebo.ab. ()

5 randomly.ab. ()

6 trial.ab. ()

7 groups.ab. ()

8 1 or 2 or 3 or 4 or 5 or 6 or 7 ()

9 back pain.tw. ()

10 exp low back pain/ ()

11 backache.tw. ()

12 lumbago.tw. ()

13 dorsalgia.tw. ()

14 lumbar vertebra* pain.mp.

15 cervical vertebra* pain.mp ()

16 thoracic vertebra* pain.mp ()

17 thoracic* pain.mp ()

18 chest pain.mp ()

19 neck pain.tw. ()

20 spin* diseas*.mp ()

21 spin* pain.mp ()

22 thoracic pain.tw ()

23 9 or 10 or 11 or 12 or 13 or 14 or 15 or 16 or 17 or 18 or 19 or 20 or 21 or 22 ()

24 manipulat*.tw. ()

25 exp Musculoskeletal Manipulations/ ()

26 high velocity low amplitude.tw. ()

27 high velocity thrust.tw. ()

28 ((spinal or joint) adj3 adjust*).tw. ()

29 mobiliz*.tw. ()

30 mobilis*.tw. ()

31 chiroprac* ()

32 osteopath* ()

33 exp musculoskeletal manipulations/ ()

34 24 or 25 or 26 or 27 or 28 or 29 or 30 or 31 or 32 or 33 ()

35 8 and 23 and 34 ()

### EMBASE (via Ovid), DATE

1 crossover-procedure/ ()

2 double-blind procedure/ ()

3 randomized controlled trial/ ()

4 single-blind procedure/ ()

5 (random* or factorial* or crossover* or cross over* or placebo* or (doubl* adj blind*) or (singl* adj blind*) or assign* or allocat* or volunteer*).mp. ()

6 1 or 2 or 3 or 4 or 5 ()

7 back pain.tw. ()

8 exp low back pain/ ()

9 backache.tw. ()

10 lumbago.tw. ()

11 dorsalgia.tw. ()

12 lumbar vertebra* pain.mp ()

13 cervical vertebra* pain.mp ()

14 thoracic vertebra* pain.mp ()

15 thoracic* pain.mp ()

16 chest pain.mp ()

17 neck pain.tw. ()

19 spin* diseas*.mp ()

19 spin* pain.mp ()

20 thoracic pain.tw ()

21 7 or 8 or 9 or 10 or 11 or 12 or 13 or 14 or 15 or 16 or 17 or 18 or 19 or 20 ()

22 manipulat*.tw. ()

23 exp musculoskeletal manipulation/ ()

24 high velocity low amplitude.tw. ()

25 high velocity thrust.tw. ()

26 ((spinal or joint) adj3 adjust*).tw. ()

27 mobiliz*.tw. ()

28 exp mobilization/ ()

29 exp joint mobilization/ ()

30 chiroprac* ()

31 osteopath* ()

32 22 or 23 or 24 or 25 or 26 or 27 or 28 or 29 or 30 or 31 ()

33 5 and 20 and 32 ()

### CENTRAL, DATE

1 (back pain):ti,ab,kw ()

2 MeSH descriptor: [Low Back Pain] explode all trees ()

3 MeSH descriptor: [Neck Pain] explode all trees ()

4 MeSH descriptor: [Spine] explode all trees ()

5 (backache):ti,ab,kw ()

6 (lumbago):ti,ab,kw ()

7 (dorsalgia):ti,ab,kw ()

8 ((lumb* near/3 pain)):ti,ab,kw ()

9 ((cerv* near/3 pain)):ti,ab,kw ()

10 #1 OR #2 OR #3 OR #4 OR #5 OR #6 OR #7 OR #8 OR #9 ()

11 (manipulat*):ti,ab,kw ()

12 MeSH descriptor: [Manipulation, Spinal] explode all trees ()

13 (high velocity low amplitude):ti,ab,kw ()

14 (high velocity thrust):ti,ab,kw ()

15 (((spinal or joint) near/3 adjust*)):ti,ab,kw ()

16 (mobiliz*):ti,ab,kw ()

17 (mobilis*):ti,ab,kw ()

18 MeSH descriptor: [Musculoskeletal Manipulations] explode all trees ()

19 #11 OR #12 OR #13 OR #14 OR #15 OR #16 OR #17 OR #18 ()

20 #10 AND #19 ()

### PEDro, DATE

1 Abstract & Title: manipulat* AND Body Part: lumbar spine, sacro-iliac joint or pelvis AND Method: clinical trial ()

2 Abstract & Title: manipulat* AND Body Part: head or neck AND Method: clinical trial ()

3 Abstract & Title: manipulat* AND Body Part: thoracic spine AND Method: clinical trial ()

### Index to Chiropractic Literature, DATE

1 Publication Type:Clinical Trial ()

2 Publication Type:Controlled Clinical Trial ()

3 Publication Type:Randomized Controlled Trial ()

4 All Fields:random* OR All Fields:placebo* OR All Fields:sham* ()

5 All Fields:clinical trial ()

6 All Fields:controlled trial ()

7 All Fields:double blind ()

8 All Fields:double-blind ()

9 All Fields:single blind ()

10 All Fields:single-blind ()

11 Publication Type:Clinical Trial OR , Publication Type:Controlled Clinical Trial OR , Publication Type:Randomized Controlled Trial OR All Fields:random* OR All Fields:placebo* OR All Fields:sham* OR All Fields:clinical trial OR All Fields:controlled trial OR All Fields:double blind OR All Fields:double-blind OR All Fields:single blind OR All Fields:single-blind ()

12 Article Title:“back pain” OR Abstract/Notes:“back pain” ()

13 Article Title:“neck pain” OR Abstract/Notes:“neck pain” ()

14 Subject:\“Low Back Pain\” ()

15 Subject:\“Neck Pain\” ()

16 All Fields:thoracic pain

17 Article Title:backache OR Abstract/Notes:backache ()

18 Article Title:lumbago OR Abstract/Notes:lumbago ()

19 Article Title:dorsalgia OR Abstract/Notes:dorsalgia ()

20 Article Title:“lumbar pain” OR Abstract/Notes:“lumbar pain” ()

21 Article Title:“lumbosacral pain” OR Abstract/Notes:“lumbosacral pain” ()

22 All Fields:cervicothoracic pain ()

23 Article Title:“back pain” OR Abstract/Notes:“back pain” OR Article Title:“neck pain” OR Abstract/Notes:“neck pain” OR Subject:\“Low Back Pain\” OR Subject:\“Neck Pain\” OR All Fields:thoracic pain OR Article Title:backache OR Abstract/Notes:backache OR Article Title:lumbago OR Abstract/Notes:lumbago OR Article Title:dorsalgia OR Abstract/Notes:dorsalgia OR Article Title:“lumbar pain” OR Abstract/Notes:“lumbar pain” OR Article Title:“lumbosacral pain” OR Abstract/Notes:“lumbosacral pain” OR All Fields:cervicothoracic pain ()

24 Article Title:manipulat* OR Abstract/Notes:manipulat* ()

25 Subject:“Manipulation, Chiropractic” ()

26 Subject:“Manipulation, Orthopedic” ()

27 Subject:“Manipulation, Osteopathic” ()

28 Subject:“Manipulation, Joint” ()

29 Subject:“Musculoskeletal Manipulations” ()

30 Article Title:“high velocity low amplitude” OR Abstract/Notes:“high velocity low amplitude” ()

31 Article Title:“high velocity thrust” OR Abstract/Notes:“high velocity thrust” ()

32 Article Title:“spinal adjustment” OR Abstract/Notes:“spinal adjustment” ()

33 Article Title:“joint adjustment” OR Abstract/Notes:“joint adjustment” ()

34 Article Title:mobiliz* OR Abstract/Notes:mobiliz* ()

35 Article Title:mobilis* OR Abstract/Notes:mobilis* ()

36 Subject:“Mobilization” ()

37 Subject:“Mobilization, Joint” ()

38 All Fields: gonstead

39 Article Title:manipulat* OR Abstract/Notes:manipulat* OR Subject:“Manipulation, Chiropractic” OR Subject:“Manipulation, Orthopedic” OR Subject:“Manipulation, Osteopathic” OR Subject:“Manipulation, Joint” OR Subject:“Musculoskeletal Manipulations” OR Article Title:“high velocity low amplitude” OR Abstract/Notes:“high velocity low amplitude” OR Article Title:“high velocity thrust” OR Abstract/Notes:“high velocity thrust” OR Article Title:“spinal adjustment” OR Abstract/Notes:“spinal adjustment” OR Article Title:“joint adjustment” OR Abstract/Notes:“joint adjustment” OR Article Title:mobiliz* OR Abstract/Notes:mobiliz* OR Article Title:mobilis* OR Abstract/Notes:mobilis* OR Subject:“Mobilization” OR Subject:“Mobilization, Joint” OR All Fields: gonstead ()

39 Article Title:pain OR Abstract/Notes:pain ()

40 Subject:“Pain” ()

41 Article Title:pain OR Abstract/Notes:pain OR Subject:“Pain” ()

42 Publication Type:Clinical Trial OR , Publication Type:Controlled Clinical Trial OR , Publication Type:Randomized Controlled Trial OR All Fields:random* OR All Fields:placebo* OR All Fields:sham* OR All Fields:clinical trial OR All Fields:controlled trial OR All Fields:double blind OR All Fields:double-blind OR All Fields:single blind OR All Fields:single-blind AND Article Title:“back pain” OR Abstract/Notes:“back pain” OR Article Title:“neck pain” OR Abstract/Notes:“neck pain” OR Subject:\“Low Back Pain\” OR Subject:\“Neck Pain\” OR All Fields:thoracic pain OR Article Title:backache OR Abstract/Notes:backache OR Article Title:lumbago OR Abstract/Notes:lumbago OR Article Title:dorsalgia OR Abstract/Notes:dorsalgia OR Article Title:“lumbar pain” OR Abstract/Notes:“lumbar pain” OR Article Title:“lumbosacral pain” OR Abstract/Notes:“lumbosacral pain” OR All Fields:cervicothoracic pain AND Article Title:pain OR Abstract/Notes:pain

## Appendix 2

### Application thrust

| Category | Choices | Details |
| --- | --- | --- |
| **Patient positioning** | Side-lying |  |
|  | Prone |  |
|  | Supine |  |
|  | Sitting |  |
|  | Standing |  |
| **Assisted** | Instrument |  |
|  | Drop-piece |  |
|  | None |  |
|  | Other |  |
| **Specific vertebral/targets** | Yes |  |
|  | No | I.e. a technique targeting multiple segments or a region of the spine |
| Region(s) target(s) | Symptomatic region | E.g. low back pain population -> only lumbar SMT used |
|  | Multiple region | Must include the symptomatic region |
|  | Non-symptomatic region | E.g. low back pain population → thoracic spine SMT used |
| **Technique** | Named system | Using a specific technique methodology (e.g., Gonstead adjustment) |
|  | No system |  |

### Choice of application site

| Category | Choices | Details |
| --- | --- | --- |
| **Selection approach** | Clinician selected | Encompasses anything where the clinician makes decisions based on any sort of assessment |
|  | Presecriptive |  |
|  | Combination | E.g. standardized target level, side based on location of pain or something else |
| **Technique system** | Yes | E.g. Activator^TM^ protocol, Gonstead assessment |
|  | No |  |
| **Rationale for selection** | Complaint history | Patient anamnesis |
|  | Palpation | E.g., static or motion palpation for either tenderness or stiffness |
|  | Asymmetry | I.e., posture (antalgia, kyphosis, lordosis, scoliosis) |
|  | Orthopedic maneuver | I.e., pain with movement that localizes region or the tissues involved |
|  | Leg Length Inequality | I.e., pelvic assessment |
|  | Range of motion | Localized regional movement |
|  | Tissue temperature, texture, tone | E.g., temperature assessment or texture-skin rolling |
|  | Specialized Tests | E.g., surface EMG, imaging, thermocouple |
|  | Combination | Any combination of the above |

*An answering option of “Not described” will be available for each aspects of the classifications*
